# Supplementary material for: Gastrointestinal Safety Assessment of GLP-1 Receptor Agonists in the US: A Real-World Adverse Events Analysis from the FAERS Database
Source: Diagnostics (Basel). 2024 Dec 16;14(24):2829. doi: 10.3390/diagnostics14242829 (PMC11675942; doi:10.3390/diagnostics14242829)
Supplement: Supplementary file 1 [file diagnostics-14-02829-s001.zip › diagnostics-3333499-supplementary.pdf]

Supplemental Table S1: Summary of FDA-approved GLP-1RAs

Generic name	Brand name	Approval date
Exenatide	Bydureon, Bydureon Bcise, Byetta	Apr. 2005
Liraglutide	Saxenda, Victoza	Jan. 2010
Dulaglutide	Trulicity	Sep. 2014
Semaglutide	Ozempic, Rybelsus	Dec. 2017

Supplemental Table S2: The top signal strength of Gastrointestinal adverse events and adverse outcomes of GLP-1 RAs ranked by number of incidence cases at the PTs level in FAERS database.

PT TERM	SOC	SOC Code	Exenatide	Liraglutide	Dulaglutide	Semaglutide
Nausea	Gastrointestinal disorders	10028896	1862	1666	1850	1996
Vomiting	Gastrointestinal disorders	10043172	839	839	946	1198
Abdominal discomfort	Gastrointestinal disorders	10005483	251	274	313	269
Abdominal pain	Gastrointestinal disorders	10001688	594	650	710	732
Pancreatitis	Hepatobiliary disorders	10030205	1174	895	486	287
Constipation	Gastrointestinal disorders	10008340	281	329	257	568
Diarrhea	Gastrointestinal disorders	10012732	744	697	995	1020
Gastrointestinal Hemorrhage	Vascular disorders	10017827	72	34	32	43
Dyspepsia	Gastrointestinal disorders	10013425	199	229	238	218
Flatulence	Gastrointestinal disorders	10014123	123	157	167	236
Delayed gastric emptying	Gastrointestinal disorders	10012657	213	210	264	326
Gastritis	Gastrointestinal disorders	10014137	49	33	15	22
GERD	Gastrointestinal disorders	10015795	117	143	125	145
Intestinal obstruction	Gastrointestinal disorders	10021163	22	26	22	23
Cholecystitis	Hepatobiliary disorders	10007410	56	26	5	19
Peptic ulcer	Gastrointestinal disorders	10037557	2	3	-	3
Anal fissure	Gastrointestinal disorders	10003536	1	1		1
Inflammatory bowel disease	Gastrointestinal disorders	10019326	14	7	4	10
**Adverse outcomes**						
Disabled	General disorders and administration site conditions	10019901	51	24	33	22
Life threatening	General disorders and administration site conditions	10021453	98	33	42	22
Hospitalization	General disorders and administration site conditions	10021454	1593	804	473	495
Death	General disorders and administration site conditions	10021455	118	36	23	15

Supplemental Table S3: Four major algorithms used for signal detection

Algorithms	Formula	Criteria
**ROR**	ROR=ad/bc<br>95%CI=$e^{\ln(ROR)\pm1.96(1/a+1/b+1/c+1/d)^{\wedge}0.5}$	a≥3, lower limit of 95% CI>1
**PRR**	PRR=a(c+d)/c/(a+b)<br>χ^2=[(ad-bc)^2](a+b+c+d)/[(a+b)(c+d)(a+c)(b+d)]	a≥3, PRR≥2, χ^2≥4,
**BCPNN**	IC=$\log_2$a(a+b+c+d)/(a+c)/(a+b)<br>95%CI=E(IC) ± 2V(IC)^0.5<br>r=(a+b+c+d)^2/(a+b+1)/(a+c+1)<br>E(IC)=$\log_2$a(a+b+c+d)^2/(a+b+c+d+r)/(a+b)/(a+c)<br>V(IC)=1/ln2(b+c+d+r-1)/(a+1)/(a+b+c+d+r+1)+(2+b+c+2d)/(a+b+1)/(a+b+c+d+r+3)<br>IC025=E(IC)- 2V(IC)^0.5	IC025>0, a≥3
**MVR**	Y = β0 + β1 x age + β2 x sex	Change in the β coefficient with p-values <0.05

Equation: a, number of reports containing both the suspect drug and the suspect adverse drug reaction; b, number of reports containing the suspect adverse drug reaction with other medications (except the drug of interest); c, number of reports containing the suspect drug with other adverse drug reactions (except the event of interest); d, number of reports containing other medications and other adverse drug reactions. ROR, reporting odds ratio; CI, confidence interval; N, the number of co-occurrences; PRR, proportional reporting ratio; χ2, chi-squared; BCPNN, Bayesian confidence propagation neural network; IC, information component; IC025, the lower limit of the 95% one-sided CI of the IC; MVR: multivariate regression, β0 is the intercept, β1 coefficient for age, and β2 coefficient for sex.
